# Progress in research on nutrition, neuroinflammation and dopaminergic alterations in Tic disorders

**DOI:** 10.3389/fped.2025.1526117

**Published:** 2025-05-20

**Authors:** Lu Bai, Mei Jin, Qi Zhang, Suzhen Sun

**Affiliations:** ^1^ Primary Child Care Department (Psychological Behavior Department), Children’s Hospital of Hebei Province, Shijiazhuang City, Hebei, China; ^2^ Department of Pediatric Neurology, Children’s Hospital of Hebei Province, Shijiazhuang City, Hebei, China; ^3^ Department of Anesthesiology, Children’s Hospital of Hebei Province, Shijiazhuang City, Hebei, China

**Keywords:** Tic disorders, dopamine, nutrition, neuroinflammation, treatment

## Abstract

Tic disorders (TD) represent a prevalent neurodevelopmental condition in children, characterised by involuntary, sudden motor or vocal tics. Dysfunction of the dopamine system plays a pivotal role in the pathogenesis of TD. Recent findings indicate that deep brain stimulation, by modulating striatal dopamine release, substantially alleviates tic symptoms. Neuroimaging studies have shown increased dopamine transporter binding and decreased serotonin levels in patients with TD. The presence of anti-dopamine D2 receptor autoantibodies, which correlate with disease severity, suggests immune involvement in the onset of TD. Nutritional factors influence the dopaminergic system's functionality by affecting neurotransmitter synthesis and metabolism, modulating gut microbiota and contributing to neuroinflammation. Clinical studies have demonstrated that interventions combining probiotics and fructooligosaccharides can help regulate neurotransmitter metabolism, whereas dietary patterns such as the ketogenic, Mediterranean and Mediterranean-DASH intervention for neurodegenerative delay diets exhibit anti-inflammatory and neuroprotective effects. The risk of TD in offspring is significantly associated with maternal autoimmune diseases and inflammatory states, with metabolic syndrome further affecting the dopaminergic system via AT1 receptor autoantibodies. Nutritional intervention-based treatment strategies present promising directions for TD management, warranting further investigation into the nutrition–immune–neurotransmitter network, the development of personalised nutritional plans and the validation of their clinical efficacy through large-scale randomised controlled trials. This review summarises the alterations in the dopaminergic system in TD, the regulatory effects of nutritional factors on dopamine levels, the interactions between neuroinflammation and the dopaminergic system and treatment strategies based on nutritional interventions, laying a theoretical foundation for understanding TD pathogenesis and advancing novel therapeutic approaches.

## 1 Introduction

Tic disorders (TD) manifest as neuropsychiatric conditions primarily characterised by involuntary, sudden motor or vocal tics. Studies indicate that TD typically begins in childhood and may co-occur with psychiatric and behavioural disorders such as attention deficit hyperactivity disorder, obsessive–compulsive disorder (OCD), anxiety disorders and depression (1). Epidemiological studies in China have reported prevalence rates of 1.7% for transient TD, 1.2% for chronic TD and 0.3% for Tourette syndrome (TS), estimating that approximately 10 million children and adolescents are affected by TD, including around 2 million cases of TS (2).

The pathogenesis of TD is multifactorial, involving genetic, immune, psychological and environmental factors. The link between its pathophysiology and clinical symptoms primarily manifests through disinhibition within the cortico-striato-thalamo-cortical loop (3). Recent research has highlighted the central role of dopaminergic system dysfunction in the pathogenesis of TD. Studies on deep brain stimulation have demonstrated that modulating striatal dopamine release can substantially alleviate tic symptoms, providing direct evidence of dopamine's key role in TD onset (4). Advanced imaging studies further reveal that patients with TD exhibit increased dopamine transporter (DAT) binding and reduced serotonin levels, underscoring the importance of neurotransmitter imbalance in disease progression (5).

Recent findings have identified anti-dopamine D2 receptor autoantibodies in patients with TD, with levels correlating to disease severity. This immunological evidence further elucidates the link between TD and dopaminergic dysfunction (6). Moreover, maternal autoimmune diseases and inflammatory states have been significantly associated with an increased risk of TD in offspring, suggesting inflammation as a critical mediator between genetic predisposition and environmental influences (7).

Nutritional factors, as modifiable environmental elements, may play a substantial role in the pathogenesis and treatment of TD. Research has shown that specific nutrients are involved in dopamine synthesis and metabolism, whereas dietary patterns can influence the gut–brain axis and neuroinflammation, thereby affecting dopaminergic system function (8). Emerging studies indicate that nutritional interventions targeting gut microbiota can influence brain dopamine levels and related behavioural manifestations (9). In addition, metabolic disorders such as obesity have been closely linked to alterations in dopamine signalling pathways, suggesting a complex interaction among the metabolic, immune and neurotransmitter systems in TD pathogenesis (10).

Conventional TD treatments primarily rely on dopamine receptor antagonists, which are often associated with notable side effects. In recent years, attention has shifted towards nutritional and anti-inflammatory treatment strategies (11). Studies have shown that certain natural compounds, such as Gastrodin, can alleviate TD symptoms by inhibiting neuroinflammation. Additionally, dietary modifications and specific nutritional supplements have emerged as promising approaches for influencing the dopaminergic system in TD therapy (12).

Given the complexity of TD pathogenesis and the limitations of current treatment options, understanding the interactions between nutrition, inflammation and the dopaminergic system is of considerable importance. This study presents a narrative review of how nutritional factors and neuroinflammation affect the dopaminergic system in TD and explores their potential therapeutic implications.

## 2 Altered function of the dopaminergic system in Tic disorders

The dopaminergic system plays a central role in the pathogenesis of TD. Advanced deep brain stimulation studies have shown that stimulation of the centromedian–parafascicular (CMPf) complex in the thalamus improves tic symptoms by regulating striatal dopamine release. In rat models, deep brain stimulation of the CMPf complex induces synaptic dopamine release and elevates baseline levels while reducing motor tic behaviours. The improvement in tic symptoms has been confirmed to be mediated by D2 receptor activation, as demonstrated through selective blockade (13).

Neuroimaging studies have found that patients with TD who are not on medication exhibit increased DAT binding in the caudate nucleus, putamen and the entire neostriatum. In patients receiving medication, DAT levels in the putamen remain normal, though increased DAT binding persists in the caudate nucleus. Additionally, there is a reduction in D2 receptor binding in the striatum and frontal cortex, accompanied by increased 5-HT2A receptor binding in the neocortex and limbic regions (14).

Research on genetic mutations further underscores the importance of the dopamine system in TD. Studies involving mice with mutations in high-confidence TD genes *CELSR3* and *WWC1* show that these mutations lead to abnormal sensorimotor behaviour, altered reward learning and increased striatal dopamine release. Prepulse inhibition tests reveal sensory gating deficits in *Celsr3* mutant mice, whereas *Wwc1* mutations produce this deficit only in women. Notably, aripiprazole—a partial agonist of the dopamine D2 receptor—corrects sensory gating deficits and abnormal upright behaviour (15).

Autoimmune responses also contribute to dopaminergic dysfunction in TD. Clinical studies indicate that 8% of patients test positive for anti-D2 receptor antibodies during symptom exacerbation, with an additional 6.6% becoming positive at later stages. Statistical analysis reveals a strong association between anti-D2 receptor antibodies and tic exacerbation (McNemar's odds ratio = 11, *p* = 0.003), a relationship that remains significant after adjusting for demographic variables and psychotropic medication use (16).

Magnetoencephalography studies reveal abnormal magnetic error-related negativity (mERN) patterns in patients with TD. Compared with controls, patients with TD do not show response-type-dependent amplitude modulation (error or correct) within 70–105 ms post-response. However, substantial mERN amplitudes are detected in both groups within the 105–160 ms window, suggesting delayed error processing in patients with TD. It is hypothesised that early error-related processing in TD is influenced by enhanced motor control triggered by the conflict between task execution and tic suppression (17).

Neuroinflammation plays a key role in dopaminergic dysfunction in TD. Experimental studies have shown that rats in a TD model induced by 1-(2,5-dimethoxy-4-iodophenyl)-2-aminopropane display pronounced neuroinflammatory responses. Elevated levels of interleukin (IL)-6, IL-1β and tumour necrosis factor-α are observed in the striatum and serum. Western blot analysis reveals activation of the TLR/NF-*κ*B and TLR/MAPK signalling pathways in the striatum (18).

These findings suggest that alterations in the dopaminergic system in TD encompass multiple aspects, including changes in neurotransmitter release, abnormal receptor expression, genetic mutations, autoimmune responses and neuroinflammation. Gaining a deeper understanding of these mechanisms is essential for developing new therapeutic strategies.

## 3 Influence of nutritional factors on dopamine levels

Nutritional status plays a crucial role in modulating brain dopamine levels. Studies have shown that gut microbiota and dietary interventions substantially influence central nervous system function. Clinical research on autism spectrum disorders has found that interventions combining probiotics and fructooligosaccharides considerably increase levels of beneficial bacteria such as *Bifidobacteria* and inhibit the growth of potential pathogens such as *Clostridium*. Following intervention, patients' serum levels of acetate, propionate and butyrate rise substantially to levels observed in control groups, whereas serum serotonin levels decrease and vanillic acid levels increase, highlighting the importance of the gut microbiota–neurotransmitter metabolic axis in neurological disease (19).

Dietary structure has a notable impact on the brain's reward system. Exposure to a high-fat diet restructures the feeding circuit and alters the motivational response to food. Animal experiments confirm that long-term high-calorie diets weaken dopaminergic neurons' response to standard food but not to high-fat food. Longitudinal recordings show that this adaptive change occurs at the level of hypothalamic agouti-related protein neurons and midbrain peripheral dopamine signalling (20).

There is considerable individual variability in the brain's response to nutritional signals. Random crossover studies have shown that, in both healthy-weight and obese individuals, intragastric glucose and lipid infusion have different effects on brain neural activity and striatal dopamine release. Healthy individuals exhibit specific neural responses and dopamine release, whereas obese individuals display severely impaired responses. Notably, diet-induced weight loss does not restore the impaired neural response in obese individuals, suggesting long-term adaptive changes in the nutrition–brain pathway (21).

Dopamine subsystems in the brain function to track homeostatic changes. Dopaminergic neurons in the ventral tegmental area (VTA) detect nutrient or water intake at different stages. Some neurons track systemic hydration changes within minutes after thirsty mice drink water, whereas others respond to nutrients in the gastrointestinal tract. Information on fluid balance, transmitted through the hypothalamic pathway, is relayed to the VTA and rerouted to downstream circuits that monitor intake stages in the mouth, gastrointestinal tract and post-absorption (22).

Dietary fat restriction also affects the activity of brain reward regions. In randomised crossover trials, selective restriction of dietary fat or carbohydrates, compared with an isocaloric baseline diet, produces different effects on dopamine D2/3 receptor binding potential and neural activity. After 5 days of fat restriction, both D2 receptor binding and neural responses to food cues in reward-related brain regions decrease, whereas no comparable changes are seen with carbohydrate restriction. Following fat restriction, spontaneous intake tends to shift towards high-fat, high-carbohydrate foods (23).

The insulin signalling pathway within the brain is closely linked to dopamine function. Studies show that central insulin action plays a key regulatory role in food intake, reward and emotional behaviours. Brain insulin resistance contributes to overeating, anxiety- and depression-like behaviours and impairs dopaminergic function. Insulin receptor sensitisers and dopamine receptor agonists have shown benefits in improving obesity and mental health conditions in both rodents and humans (24).

## 4 Interactions between neuroinflammation and the dopaminergic system

Neuroinflammation plays a pivotal role in the pathogenesis of neuropsychiatric disorders. Maternal autoimmune diseases and inflammatory states have been shown to be closely associated with the onset of TD and OCD in children. Studies report that the incidence of autoimmune diseases in mothers of children with TD is higher than in mothers of children with other autoimmune neurological disorders (*p* = 0.054) and significantly higher than in healthy control mothers (*p* = 0.0004). Furthermore, the incidence of autoimmune diseases is also markedly elevated among first- and second-degree maternal relatives of children with TD (*p* < 0.0001 and *p* = 0.014, respectively). Transcriptome analysis indicates that differentially expressed genes upregulated in the brains of patients with TD and maternal autoimmune disease are enriched in innate immune processes (25).

Metabolic syndrome exerts its effects on the dopaminergic system via mediation by AT1 receptor autoantibodies. Experimental data suggest that metabolic syndrome enhances activity of the pro-inflammatory renin–angiotensin system axis in the substantia nigra, leading to increased oxidative stress and neuroinflammation, which in turn exacerbate dopaminergic neuron degeneration. Administration of AT1 receptor blockers has been shown to mitigate these pathological changes. In rats with metabolic syndrome, serum levels of LIGHT and other key pro-inflammatory cytokines, as well as 27-hydroxycholesterol, are elevated alongside a marked increase in pro-inflammatory AT1 and ACE2 autoantibodies (26).

The dopaminergic pathway is also critical in the context of obesity-related inflammation. Research has found that dopamine not only regulates immune function but is also produced endogenously by immune cells. In the nigrostriatal and mesocorticolimbic systems, the inflammatory environment alters dopaminergic signalling by promoting the release of pro-inflammatory cytokines (including IL-1β, IL-6 and TNF-α). These inflammatory factors, once in systemic circulation, initiate widespread inflammation that further affects brain function and dopamine signalling (27).

Metabolic syndrome and obesity affect the occurrence, symptomatic presentation and progression of TD through a variety of mechanisms. Studies have shown that obesity-related chronic low-grade inflammatory states may lead to dysfunction of the brain's dopaminergic system, thereby promoting the development of TD or worsening its symptoms (3). For example, hyperprolactinaemia promotes weight gain, obesity and metabolic syndrome by inhibiting physiological dopaminergic tone and impairing glucose–insulin and lipid metabolism (14). This endocrine metabolic disorder not only affects overall health but may also indirectly influence TD symptoms by altering dopamine levels in the brain. In addition, insulin resistance associated with metabolic syndrome may affect the pathological process of TD by interfering with dopamine signalling (17). Regulating these metabolic abnormalities may represent a new direction in the treatment of TD.

Regulation of inflammatory responses also presents a promising avenue for TD management. Gastrodin, an active constituent of traditional Chinese medicine, is widely used for its sedative, anticonvulsant and neuroprotective effects. In TD animal models, gastrodin has been shown to substantially reduce abnormal stereotypic behaviours by decreasing the number of D2 receptors and increasing DT expression. Additionally, gastrodin reduces serum transporter density, thereby indirectly lowering dopamine release (28).

Combined treatment with glucagon-like peptide-1 (GLP-1) and nicotine has been shown to improve obesity by acting on the hypothalamic and mesocorticolimbic pathways. Studies report that the combination of the GLP-1 receptor agonist liraglutide with nicotine suppresses food intake and increases energy expenditure, resulting in reduced body weight in obese mice. This drug combination activates multiple brain areas, with liraglutide enhancing the excitability of hypothalamic pro-opiomelanocortin neurons and VTA dopaminergic neurons. Using genetically encoded dopamine sensors, researchers have shown that liraglutide can inhibit nicotine-induced dopamine release in the nucleus accumbens of freely behaving mice (29).

Brain insulin action also interacts with the dopamine system in the context of schizophrenia. Studies have shown that central nervous system insulin is involved in regulating striatal dopamine levels, peripheral glucose homeostasis and feeding behaviour. Insulin receptors and dopamine D2 receptors are co-expressed on human pancreatic β-cells and adipocytes, supporting the critical role of both insulin and dopamine in peripheral metabolic regulation. Clinical evidence confirms that dopaminergic drugs can improve the clinical manifestations of metabolic syndrome and obesity, substantially enhancing glucose–insulin metabolism and lipid profiles (30).

## 5 Nutritional intervention-based treatment strategies

Nutritional interventions have demonstrated notable efficacy in the treatment of neurological diseases. The ketogenic diet (KD) is well established as a therapy for refractory epilepsy and is currently under investigation for its potential use in febrile infection-related epilepsy syndrome and epileptic encephalopathies. Research has substantiated that KD acts by specifically targeting dysregulated adaptive and innate immune responses in refractory epilepsy and status epilepticus. The diet has also shown anti-inflammatory and neuroprotective effects in models of multiple sclerosis, Parkinson's disease, pain and spinal cord injury. Ketone bodies, caloric restriction, polyunsaturated fatty acids and alterations in gut microbiota all contribute to KD's anti-inflammatory effects (31).

The Mediterranean diet (MD) has shown substantial efficacy in the secondary prevention of cardiovascular disease. Results from randomised controlled trials indicate that, compared with a low-fat diet, the MD is associated with a reduced incidence of major cardiovascular events. After multivariate adjustment, the hazard ratio ranged from 0.719 to 0.753, consistently favouring the MD group. The effect was more pronounced in men, with a major endpoint event rate of 16.2% in the Mediterranean group compared with 22.8% in the low-fat group (multifactor-adjusted HR 0.669, *p* = 0.013). Research has also confirmed that the MD enhances neurological function through anti-inflammatory and neuroprotective mechanisms (32).

The Mediterranean-DASH intervention for neurodegenerative delay (MIND) diet combines features of the Mediterranean and DASH diets. In a randomised controlled trial, 604 elderly individuals without cognitive impairment but with a family history of dementia were assigned to either the MIND diet or a control diet with caloric restriction. After a 3-year follow-up, both groups showed improvements in global cognitive function scores, with the MIND diet group reporting an increase of 0.205 standard units and the control group 0.170. MRI results showed comparable changes in white matter hyperintensities, hippocampal volume and total grey and white matter volumes between the groups (33).

Dietary interventions in Parkinson's disease research indicate that protein-restricted diet, KD, MD and MIND diet all influence disease risk, progression and severity. Protein-restricted diet primarily improves medication absorption by reducing competition between levodopa and dietary protein. Ketogenic diet provides an alternative energy source and exerts neuroprotective effects through its anti-inflammatory action. Mediterranean diet, rich in antioxidants and omega-3 fatty acids, helps mitigate neurodegenerative changes (34).

Research on nutritional interventions for cognitive decline in elderly individuals suggests that changes in dietary patterns can have a protective effect on brain health. Anti-inflammatory diets—particularly the Mediterranean, Okinawan and MIND diets—have been shown to support nervous system health. Omega-3 fatty acids, antioxidants and polyphenolic compounds inhibit neuroinflammation associated with Alzheimer's disease. Additionally, anti-inflammatory diets reduce neuroinflammation through indirect immune pathways involving the gut microbiota and systemic circulation (35).

The influence of dietary patterns on migraines has been extensively investigated. Clinical observations have shown that certain dietary factors can trigger or exacerbate migraine attacks. Dietary intervention strategies include elimination diets, ketogenic diets and comprehensive diets. Despite inconsistencies in the literature and a lack of consensus, existing data support the potential benefits of dietary interventions for some patients with migraine. Factors such as age, gender, genetics and environmental conditions all play a role in determining the outcomes (36).

## 6 The influence of nutritional factors on the occurrence and development of Tic disorders

Nutritional status is closely related to the pathophysiology of TD. Studies have shown that specific nutrients can affect TD by modulating neuroinflammation, improving gut microbiota composition and directly influencing dopamine metabolism pathways in the brain. For example, antioxidants and omega-3 fatty acids are thought to reduce neuroinflammation, which may lower the risk of TD or help alleviate its symptoms (34). In addition, certain dietary patterns, such as the MD and the MIND diet, not only support overall health but may also be particularly beneficial for patients with TD by optimising environmental stability within the brain and enhancing neuroprotection (32, 33).

Recent studies have shown that adjusting the diet to include more beneficial ingredients can be used as an adjunct therapy to ease symptoms and improve the quality of life for people with TD. One study found that the use of probiotics and fructooligosaccharide interventions in the TD model could modulate the gut microbiota, which in turn affected serum neurotransmitter levels through the gut–brain axis—including a decrease in serum serotonin levels and an increase in vanillic acid levels—providing a new perspective on the role of nutritional factors in TD pathophysiology (9).

Research on the effectiveness of nutritional interventions for TD is still in its early stages, but there is evidence that some dietary patterns may offer therapeutic value. For example, the KD, as a high-fat, low-carbohydrate diet, has shown anti-inflammatory and neuroprotective effects in various neurological disorders. Although fewer studies have directly focused on TD, its positive effects in similar conditions suggest potential for future research (23). In addition, it has been shown that Gastrodin, a traditional Chinese medicine ingredient, can reduce TD symptoms by inhibiting neuroinflammatory pathways (11, 21). Although most of these findings are based on animal models, they provide a theoretical foundation for developing nutrition-based treatments.

In summary, although the current understanding of how nutritional factors specifically affect TD is still incomplete, existing research suggests that dietary modification and the introduction of specific nutrient supplements may be an effective strategy to improve TD symptoms. Further clinical trials are needed to validate these findings and to better elucidate the role of nutritional factors in TD management (Table 1).

**Table 1:** Effects of nutritional factors on the dopaminergic system and its mechanisms.

Factors	Influence mechanism	Effects on the dopaminergic system	Relevant research/literature
Antioxidants and omega-3 fatty acids	Reduces neuroinflammation and improves overall health	May reduce the risk of TD or reduce its symptoms	[Wang Y, et al., (9) 2020]
Probiotics and fructooligosaccharides	Regulates the gut microbiota and influences neurotransmitter levels through the gut-brain axis	Improves serum neurotransmitter levels (e.g., lowers 5-HT, increases vanillic acid)	[Wang Y, et al., (9) 2020]
Mediterranean diet/MIND diet	Improves the stability of the environment in the brain and enhances neuroprotection	Improve the quality of life of people with TD	[Dyńka D, et al., (23) 2022]
High-fat diet	Leads to the reorganization of feeding circuits, altering the food response of the motivational system	Impairs dopaminergic neuronal responses to standard foods, but not high-fat foods	[Mazzone CM, et al., (32) 2020]
Gastrodin	Inhibits neuroinflammatory pathways	Reduces TD symptoms, reduces the number of D2 receptors, and enhances dopamine transporter expression	[Long H, et al., (11) 2019; Wang Y, et al., (9) 2021]

## 7 Effect of neuroimmune factors on Tic disorders

Neuroimmune factors influence the development of TD through a variety of mechanisms. Studies have shown that the autoimmune or inflammatory state experienced by the mother during pregnancy is associated with an increased risk of TD in offspring (4). This type of immune activation may lead to abnormal brain development during the fetal period, particularly in regions involving the dopaminergic system, thereby increasing the risk of TD later in life. Additionally, the presence of anti-dopamine D2 receptor antibodies may be associated with chronic TD, suggesting that immune-mediated mechanisms play an important role in the pathophysiology of TD (6).

Neuroimmune factors not only affect the onset of TD but also have a substantial impact on symptom expression. For instance, insulin resistance associated with metabolic syndrome may influence the symptomatic presentation of TD by interfering with the dopamine signalling pathway (17). Inflammatory mediators such as IL-1β, IL-6 and TNF-α can exacerbate neuroinflammation and alter dopaminergic function in the brain, thereby worsening TD symptoms (32). This inflammatory state not only affects localised brain regions but can also spread systemically through blood circulation, further impacting overall health. As the condition progresses, neuroimmune factors continue to play a key role. Long-term exposure to high levels of inflammatory cytokines may lead to the degeneration of dopaminergic neurons, a major factor in the exacerbation of TD (3).

Regarding prognosis, modulating the inflammatory response is considered a potential strategy to improve long-term outcomes. Gastrodin, a traditional Chinese medicine ingredient, has been shown to substantially reduce aberrant behaviour by decreasing D2 receptor numbers and enhancing DT expression in animal models of TD (11, 21). It also reduces serum transporter density and indirectly lowers dopamine release, helping to alleviate TD symptoms and improve overall outcomes.

In summary, neuroimmune factors influence the onset, symptomatology, progression and prognosis of TD through a range of complex mechanisms. Understanding these processes not only aids in the development of new treatments but also provides a theoretical foundation for personalised medicine. Future research should focus more closely on the interaction between neuroimmunity and the dopaminergic system to improve the management of TD (Table 2).

**Table 2:** Effects of neuroinflammation on the dopaminergic system and its mechanisms.

Factors	Influence mechanism	Effects on the dopaminergic system	Relevant research/literature
Maternal autoimmune disease	Affects fetal brain development	Increase the risk of TD in future generations	[Jones HF, et al., (4) 2021]
AT1 receptor autoantibodies	Enhances the activity of the renin-angiotensin system, leading to oxidative stress and neuroinflammation	Exacerbates dopaminergic neuronal degeneration	[Pedrosa MA, et al., (17) 2023]
Inflammatory mediators (IL-1β, IL-6, TNF-α)	Increases the production of pro-inflammatory cytokines, triggering systemic inflammation	Alter dopaminergic signaling and worsen TD symptoms	[Nikolaus S, et al., (3) 2022]

## 8 Prospects for future studies

Further exploration is needed to integrate nutritional interventions with immune modulation in treatment strategies for nervous system diseases. Current research indicates that the KD improves neurological function by modulating metabolism and the immune system. The KD has shown promising therapeutic effects in refractory epilepsy, multiple sclerosis and neurodegenerative diseases. Mechanistic studies suggest that its anti-inflammatory action is mediated by multiple factors, including ketone bodies, caloric restriction, polyunsaturated fatty acids and changes in gut microbiota composition (37).

The role of the gut microbiota–metabolite–brain axis in nervous system diseases warrants in-depth investigation. A low-protein, high-carbohydrate diet has demonstrated neuroprotective effects in MPTP-induced Parkinson's disease mouse models. This dietary intervention alters gut microbiota composition, increasing beneficial bacteria such as *Bifidobacterium* and *Ileibacterium*, whereas reducing the abundance of *Bilophila* and *Alistipes*. PICRUSt-predicted faecal microbiome functions indicate that this diet suppresses lipopolysaccharide biosynthesis and the tricarboxylic acid cycle while enhancing amino acid and carbohydrate metabolism (38).

Deoxyribonucleic acid methylation, as an epigenetic modification, is influenced by both metabolic and nutritional factors. Studies have shown that bioactive nutrients and gut microbiota can alter DNA methylation in the central nervous system via the gut–brain axis, thereby affecting neural function and behaviour. Deoxyribonucleic acid hydroxymethylation, in particular, is prevalent in the adult brain, and dietary interventions aimed at modulating this epigenetic process represent a novel therapeutic approach (39).

Studies on nutritional and exercise interventions for patients with sarcopenia and obesity offer novel insights into improving metabolic health. Meta-analyses indicate that aerobic exercise reduces body weight and fat mass, resistance exercise reduces fat mass and improves grip strength and their combination reduces fat mass and enhances walking speed. Nutritional interventions, particularly low-calorie, high-protein diets, reduce fat mass without affecting muscle mass or grip strength. However, nutritional supplementation combined with exercise does not confer additional benefits (40).

Endocrine metabolic disorders are closely linked to the neurotransmitter system. Hyperprolactinaemia promotes weight gain, obesity and the development of metabolic syndrome by inhibiting physiological dopaminergic tone and disrupting glucose–insulin and lipid metabolism. Human pancreatic β-cells and adipocytes express both prolactin receptors and dopamine D2 receptors, highlighting the key role of prolactin and dopamine in peripheral metabolic regulation. Dopamine receptor agonists, including bromocriptine and cabergoline, have been shown to reduce the prevalence of metabolic syndrome and obesity while improving metabolic indicators (41).

The brain's reward system plays a vital role in metabolic regulation. Dopamine neurons in the VTA track internal state changes and respond to nutrients at various stages of ingestion. Hypothalamic pathways transmit fluid balance information to the dopamine system, which redistributes it to downstream circuits that monitor oral, gastrointestinal and post-absorptive stages. A deeper understanding of the relationship between the reward system and metabolic regulation may offer new approaches for treating metabolic diseases (42).

## 9 Conclusion

The dopaminergic system's pivotal role in TD, and its intricate interplay with nutritional factors and neuroinflammation, has been highlighted by recent research. Deep brain stimulation studies have validated the mechanism of improving tic symptoms by modulating striatal dopamine release. Neuroimaging studies have identified abnormal changes in DT and receptor expression in patients with TD, whereas genetic studies have clarified the connection between high-confidence TD genes and dopaminergic system functionality. Nutritional factors influence the dopaminergic system by affecting neurotransmitter synthesis and metabolism, modulating gut microbiota and contributing to neuroinflammation. The association between maternal immune-inflammatory states and increased TD risk in offspring underscores the importance of immune-inflammatory factors in disease occurrence. These findings suggest that nutritional interventions may offer a novel therapeutic approach for TD. Specific dietary patterns, including the KD, MD and MIND diet, have been shown to improve neurological function through anti-inflammatory and neuroprotective effects. Future research should explore the detailed mechanisms underlying the nutrition–immune–neurotransmitter network, develop personalised nutritional intervention plans and investigate the synergistic effects of nutritional strategies alongside existing treatment methods. Further studies into the gut microbiota–metabolite–brain axis, the regulation of DNA methylation and the relationship between the reward system and metabolic regulation may reveal new mechanisms of TD pathogenesis and provide potential targets for prevention and treatment. Long-term, large-scale randomised controlled trials are essential for validating the clinical efficacy of nutritional interventions and clarifying their mechanisms of action.
